# 血清肿瘤标志物与一线EGFR-TKIs治疗晚期*EGFR*突变型肺腺癌患者疗效相关性分析

**DOI:** 10.3779/j.issn.1009-3419.2017.09.01

**Published:** 2017-09-20

**Authors:** 含笑 陈, 雪 杨, 慧君 刘, 琨 马, 佳 仲, 智 董, 明磊 卓, 玉艳 王, 俭杰 李, 彤同 安, 梅娜 吴, 子平 王, 军 赵

**Affiliations:** 1 100142 北京，北京大学肿瘤医院暨北京市肿瘤防治研究所胸部肿瘤内一科，恶性肿瘤发病机制及转化研究教育部重点实验室 Key laboratory of Carcinogenesis and Translational Research (Ministry of Education/Beijing), Department of Thoracic Oncology, Peking University Cancer Hospital & Institute, Beijing 100142, China; 2 030000 太原，西山煤电职工总医院肿瘤科 Department of Oncology, General Hospital of Xishan Coal and Electricity Workers, Taiyuan 030000, China; 3 067000 承德，承德市承钢医院肿瘤科（二内科） Department of Oncology, Chenggang Hospital, Chengde 067000, China

**Keywords:** 外周血肿瘤标志物, 肺肿瘤, EGFR酪氨酸激酶抑制剂, 疗效, 生存, Serum tumor markers, Lung neoplasms, EGFR-TKIs, Efficacy, Survival

## Abstract

**背景与目的:**

表皮生长因子受体酪氨酸激酶抑制剂（epidermal growth factor receptor tyrosine kinase inhibitors, EGFR-TKIs）在*EGFR*突变型肺腺癌人群中能显著提高生存，从而改变了晚期肺癌的治疗模式，但并不是所有EGFR敏感突变者均能从EGFR-TKIs治疗中获益。本研究欲通过外周血肿瘤标志物的检测对突变型肺腺癌患者的靶向治疗进行预测及指导。

**方法:**

回顾性分析2009年6月-2014年6月于北京大学肿瘤医院胸部肿瘤内一科一线接受EGFR-TKIs治疗的*EGFR*突变型Ⅲb期-Ⅳ期肺腺癌患者的临床资料，分析其基线肿瘤标志物与EGFR-TKIs疗效及生存的关系。

**结果:**

总体人群客观有效率（objective response rate, ORR）52.8%，疾病控制率（disease control rate, DCR）89.3%。基线癌胚抗原（carcino-embryonic antigen, CEA）水平升高者对EGFR-TKIs疗效更佳（ORR 61.3% *vs* 35.9%, DCR 95.2% *vs* 74.4%, *P* < 0.001），治疗1个月后CEA、细胞角蛋白19片段（cytokeratin 19 fragments, CYFRA21-1）以及CA125水平下降者有效率更高（ORR分别是61.5% *vs* 25%，*P*=0.002；58.5% *vs* 37.5%，*P*=0.004；61.8% *vs* 20%，*P*=0.027）。生存分析中，基线CEA水平正常者较高水平者无进展生存期（progression-free survival, PFS）明显缩短（中位PFS 5.9个月*vs* 9.8个月，*P*=0.027），而基线CYFRA21-1、CA125水平升高者PFS明显缩短（中位PFS 9.0个月*vs* 11.4个月，*P*=0.029；9.0个月*vs* 11.5个月，*P*=0.023）。多因素分析显示，美国东部肿瘤协作组（Eastern Cooperative Oncology Group, ECOG）评分0-1分、基线CYFRA21-1正常水平、治疗1月后CEA下降阳性患者PFS更长。总生存期（overall survival, OS）与CYFRA21-1、CA125升高有关（中位OS分别为25.1个月*vs* 52.5个月，*P*=0.003；22.7个月*vs* 55.0个月，*P* < 0.001），而多因素分析中总生存与CEA下降有关（*P*=0.046）。

**结论:**

治疗前高水平CEA以及治疗后CEA下降可以预测晚期肺腺癌患者一线接受EGFR-TKIs的疗效，而治疗前高水平CYFRA21-1以及CA125则预示着生存期缩短。

## Abstract (en)

**Background and objective:**

Epidermal growth factor receptor tyrosine kinase inhibitors (EGFR-TKIs) significantly improve the survival of advanced lung adenocarcinoma patients harboring *EGFR* mutation. Limited to the standards of tumor tissue samples and detection methods, still some people can't receive target therapy following genetic guidance. This study was to explore the relevance between serum tumor markers and treatment of EGFR-TKIs.

**Methods:**

We retrospectively collected the clinical information of advanced lung adenocarcinoma patients harboring *EGFR* mutation, who received EGFR-TKIs as first-line therapy from June 2009 to June 2014 in Peking University Cancer Hospital, analyzed the relationship between serum tumor markers and efficacy of EGFR-TKIs.

**Results:**

The objective response rate (ORR) was 52.8% and the disease control rate (DCR) was 89.3%. The results showed that, patients with high CEA level before treatment responded better to TKIs (ORR 61.3% *vs* 35.9%, DCR 95.2% *vs* 74.4%, *P* < 0.001). Similar phenomena was found in patients with CEA decreased 1 month later (61.5% *vs* 25%, *P*=0.002). Progression-free survival (PFS) significantly prolonged in patients with elevated baseline CEA (mPFS 9.8 mo *vs* 5.9 mo, *P*=0.027). To the opposite, PFS was significantly shorter in patients with elevated baseline CYFRA21-1 and CA125 (mPFS 9.0 mo *vs* 11.4 mo, *P*=0.029; 9.0 mo *vs* 11.5 mo, *P*=0.023, respectively). Multivariate analysis showed that Eastern Cooperative Oncology Group (ECOG) score of 0-1, normal baseline CYFRA21-1 and CEA decline predicted longer PFS. The overall survival (OS) was highly associated with elevated CYFRA21-1 and CA125 (median OS 25.1 mo *vs* 52.5 mo, *P*=0.003; 22.7 mo *vs* 55.0 mo, *P* < 0.001, respectively), while independent of CEA.

**Conclusion:**

High level of baseline CEA and decline 1 month after treatment could predict the efficacy of EGFR-TKIs in patients with advanced lung adenocarcinoma. While high levels of baseline CYFRA21-1 and CA125 indicated shortened survival.

肺癌目前仍是恶性肿瘤中发病率及死亡率领先的肿瘤，大部分肺癌诊断时已为晚期^[1]^，中位生存时间不到1年^[2]^。表皮生长因子受体酪氨酸激酶抑制剂（epidermal growth factor receptor tyrosine kinase inhibitors, EGFR-TKIs）在*EGFR*基因突变型非小细胞肺癌（non-small cell lung cancer, NSCLC）人群中能显著提高其疗效及生存，从而改变了晚期肺癌的治疗模式^[3-7]^，但IPS0S（益普索集团）2014年的调查报告显示我国*EGFR*基因检测率只有27%，而且即便是*EGFR*突变型，也并非所有患者均能获益，因此，我们仍需要更多经济、便捷的指标来挑选优势人群，以指导EGFR-TKIs的应用。

外周血肿瘤标志物是由机体对肿瘤反应或肿瘤组织自身产生的肽类物质，可反映肿瘤状态，由于其检测方法方便、经济、创伤小，有望成为预测或监测治疗疗效的标志^[8]^。既往研究发现，CEA、CYFRA21-1等肿瘤标志物与TKIs疗效有一定相关性^[9-12]^，但大部分研究中患者基因突变状态、既往治疗等条件未经选择，导致部分研究结果受影响。本研究回顾性分析了多项血肿瘤标志物与晚期*EGFR*突变型肺腺癌患者一线接受EGFR-TKIs治疗的疗效及生存的相关性，以期判断疾病的预后，并指导部分患者的治疗。

## 1 对象与方法

### 1.1 研究对象

选取2009年6月-2014年6月于北京大学肿瘤医院胸部肿瘤内一科一线接受EGFR-TKI治疗的*EGFR*突变型Ⅲb期-Ⅳ期肺腺癌患者，收集性别、年龄、吸烟史、初诊分期[国际抗癌联盟（Union for International Cancer Control, UICC）第7版NSCLC肿瘤-淋巴结-转移（tumor-node-metastasis, TNM）分期]（临床或病理分期）、美国东部肿瘤协作组（Eastern Cooperative Oncology Group, ECOG）评分、靶向治疗前及治疗1个月后肿瘤标志物水平。

### 1.2 研究方法

#### 1.2.1 基因检测

收集患者石蜡包埋组织或切片，使用*EGFR*基因检测试剂盒（艾德，厦门），采取突变扩增阻滞系统（ARMS）法，对EGFR 18、19、20、21外显子进行突变检测，根据试剂盒判断标准确定*EGFR*基因突变状态。

#### 1.2.2 外周血肿瘤标志物检测

外周血肿瘤标志物由北京肿瘤医院检验科进行检测，项目包括CEA（正常值0-5 ng/mL）、NSE（正常值0-15.2 ng/mL）、SCC（0-1.5 ng/mL）、CYFRA21-1（0-3.3 ng/mL）、CA125（0-35 U/mL）以及CA199（0-37 U/mL），超过正常值上限为阳性结果。

#### 1.2.3 治疗方案

纳入患者一线治疗均为EGFR-TKIs，包括厄洛替尼（特罗凯，150 mg *Qd*）、吉非替尼（易瑞沙，250 mg *Qd*）或埃克替尼（凯美纳，125 mg *Tid*）。

#### 1.2.4 二代测序

13例患者在治疗前后治疗进展后送检组织（5例）或外周血（7例）、胸水（1例）的标本（北京基因加公司科技有限公司），采用IlluminaNextSeq CN500基因测序平台，结合ER-Seq测序方法，进行二代测序。

### 1.3 疗效评价

靶向治疗1月后进行首次复查，随后每2月复查，包括胸部增强计算机断层扫描（computed tomography, CT）以及转移部位影像学检查。按照实体瘤客观疗效评价标准实体瘤疗效评价标准（Response Evaluation Criteria in Solid Tumors, RECIST）1.0版进行疗效评估，分为完全缓解（complete response, CR）、部分缓解（partial response, PR），疾病稳定（stable disease, SD）及疾病进展（progressive disease, PD），计算客观缓解率（objective response rate, ORR）及疾病控制率（disease control rate, DCR）。无进展生存期（progression free survival, PFS）定义为治疗开始到疾病进展或死亡的时间，总生存期（overall survival, OS）定义为疾病诊断到死亡的时间。

### 1.4 统计学方法

采用IBM SPSS 19.0统计学软件。上述临床病理特征及肿瘤标志物与EGFR-TKIs治疗疗效的关系采用*Pearson* χ^2^检验。生存分析采用*Kaplan-Meier*方法，计算中位PFS、OS及其95%可信区间（95% confidence interval, 95%CI）。多因素分析采用*Cox*比例风险回归模型，计算优势比（odds ratio, OR）及其95%CI。采用双侧检验，*P* < 0.05为差异有统计学意义。

## 2 结果

### 2.1 患者特征

本研究共入组208例患者，其中男性80例，女性128例，中位年龄61岁，123例为*EGFR*基因19外显子缺失突变，85例为21外显子L858R突变。上述患者具体临床病理特征及与EGFR-TKI疗效相关性见表 1。其中治疗前CEA升高者75.4%（126/167），NSE升高者64.1%（100/156），SCC升高者7.6%（11/145），CYFRA21-1升高者67.7（105/155），CA125升高者63.6%（91/143），CA199升高者27.8%（40/144）。

**1:** 患者临床病理特征及与EGFR-TKIs疗效相关性 Patient characteristics and correlation with EGFR-TKIs

Characteristic	*n* (%)	Efficacy [*n* (%)]			*P*
		CR/PR	SD	PD	
Gender					0.064
Male	80 (31.5)	42 (53.8)	31 (39.7)	5 (6.4)	
Female	128 (68.5)	49 (39.2)	58 (46.4)	18 (14.4)	
Age					0.34
< 65 yrs	135 (64.9)	57 (43.5)	56 (42.7)	18 (13.7)	
≥65 yrs	73 (35.1)	34 (47.2)	33 (45.8)	5 (6.9)	
Smoking history					0.11
Ever	51 (24.5)	64 (41.8)	68 (44.8)	21 (13.7)	
No	157 (75.5)	27 (54.0)	21 (42.0)	2 (4.0)	
ECOG score					0.051
0-1	187 (91.7)	85 (46.2)	81 (44.0)	18 (9.8)	
2-3	17 (8.3)	5 (29.4)	7 (41.2)	5 (29.4)	
TNM stage					0.38
Ⅲb	9 (4.2)	5 (55.6)	2 (22.2)	2 (22.2)	
Ⅳ	205 (95.8)	101 (49.3)	83 (40.5)	21 (10.2)	
Metastatic sites					0.50
< 3	137 (69.5)	59 (43.7)	63 (46.7)	13 (9.6)	
≥3	60 (30.5)	29 (48.3)	23 (38.3)	8 (13.3)	
*EGFR* mutation					0.48
19 exon deletion	123 (59.1)	52 (43.0)	57 (47.1)	12 (9.9)	
21 exon	85 (40.9)	39 (47.6)	32 (39.0)	11 (13.4)	
ECOG: Eastern Cooperative Oncology Group; EGFR: epidermal growth factor receptor; TNM: tumor-node-metastasis; CR: complete response; PR: partial response; SD: stable disease; PD: progressive disease.					

### 2.2 肿瘤标志物与EGFR-TKIs疗效的关系

#### 2.2.1 靶向治疗前肿瘤标志物水平与EGFR-TKIs疗效的关系

208例经过影像学评效的患者中，总体人群有效率52.8%（110/208），疾病控制率89.3%（186/208）。靶向治疗前CEA水平升高者较CEA正常者更能从EGFR-TKIs治疗中获益（ORR 61.3% *vs* 35.9%, DCR 95.2% *vs* 74.4%, *P* < 0.001）。随着CEA的升高，分别以超出正常值上限10倍、20倍为界，这种治疗获益仍然存在，ORR分别为60% *vs* 39.3%、61.1% *vs* 41.9%，DCR分别为95.3% *vs* 80.3%、95.8% *vs* 82.4%，*P*=0.022、*P*=0.042。

其他肿瘤标志物如NSE、SCC、CYFRA21-1、CA125、CA199与EGFR-TKIs疗效之间并无明显相关性，其中，虽然无统计学差异，CA125的升高一定程度上提高了TKIs的疗效（ORR 56.2% *vs* 40.0%, *P*=0.068）（表 2）。

**2:** 肿瘤标志物与EGFR-TKIs疗效相关性 Correlation between serum tumor markers and efficacy of EGFR-TKIs

Serum tumor markers	Efficacy (high level vs normal)		*P*
	ORR (%)	DCR (%)	
CEA	61.3 *vs* 35.9	95.2 *vs* 74.4	< 0.001
CEA decreased 1 mo later	62.2 *vs* 50.0	97.3 *vs* 88.5	0.014
NSE	47.5 *vs* 52.8	90.9 *vs* 90.6	0.45
NSE decreased 1 mo later	52.6 *vs* 45.0	97.4 *vs* 90.2	0.26
SCC	55.6 *vs* 47.5	100.0 *vs* 91.2	0.65
SCC decreased 1 mo later	80.0 *vs* 50.0	100.0 *vs* 100.0	0.09
CYFRA21-1	51.9 *vs* 44.7	90.4 *vs* 91.5	0.63
CYFRA21-1 decreased 1 mo later	58.5 *vs* 37.5	97.6 *vs* 62.5	0.004
CA125	56.2 *vs* 40.0	92.1 *vs* 96.0	0.068
CA125 decreased 1 mo later	61.8 *vs* 20.0	97.1 *vs* 60.0	0.027
CA199	45.7 *vs* 47.4	88.6 *vs* 94.7	0.46
CA199 decreased 1 mo later	60.0 *vs* 0.0	93.6 *vs* 66.7	0.25
Multiple elevated tumor markers	52.5 *vs* 49.5	96.6 *vs* 85.3	0.08
ORR: objective response rate; DCR: disease control rate; EGFR-TKIs: EGFR-tyrosine kinase inhibitors.			

#### 2.2.2 靶向治疗前多项肿瘤标志物升高与EGFR-TKIs疗效的关系

本研究中将2项及以上肿瘤标志物升高定义为多项肿瘤标志物升高，发现多项肿瘤标志物升高者与单项肿瘤标志物升高者疗效相当（ORR 52.2% *vs* 48.3%, DCR 93.5% *vs* 84.5%, *P*=0.2）。进一步根据肿瘤标志物升高个数进行分组（1-2/6; 3-4/6; 5-6/6），发现6项肿瘤标志物中，越多肿瘤标志物升高，EGFR-TKIs疗效可能更好，但这种差异无统计学差异（三组ORR分别为47.3% *vs* 45.5% *vs* 61.1%，DCR分别是83.6% *vs* 92.4% *vs* 94.4%，*P*=0.34）。

#### 2.2.3 治疗前后肿瘤标志物的变化与EGFR-TKIs疗效的关系

在203例患者中，58例患者在治疗后1月复查肿瘤标志物，比较治疗前后肿瘤标志物的变化，发现CEA下降的患者TKIs疗效更好（ORR 61.5% *vs* 25%, *P*=0.002）。同样的，TKIs治疗后CA125、CYFRA21-1下降的患者明显疗效更好（CA125: ORR 61.8% *vs* 20.0%, *P*=0.027; CYFRA21-1: ORR 58.5% *vs* 37.5%, *P*=0.004），而在其他肿瘤标志物（NSE、SCC以及CA199）中并未发现上述差异（表 2）。

### 2.3 肿瘤标志物与生存的关系

整体人群中位PFS 9.5个月。单因素分析，基线CEA水平正常者EGFR-TKIs治疗PFS明显短于CEA正常者（中位PFS 5.9个月*vs* 9.8个月，*P*=0.027）（图 1A），与之相反的是，基线NSE、CYFRA21-1、CA125水平升高者较肿瘤标志物正常者PFS明显缩短（中位PFS分别是7.9个月*vs* 11.5个月，*P*=0.015；9.0个月*vs* 11.4个月，*P*=0.029；9.0个月*vs* 11.5个月，*P*=0.023）（图 1B-图 1D）。而多项肿瘤标志物升高者PFS明显缩短（中位PFS 8.1个月*vs* 15.3个月，*P*=0.03）（图 1E），因CEA水平与PFS呈明显正相关，将其去除，仍可见多项肿瘤标志物升高者PFS缩短（中位PFS 8.9个月*vs* 9.7个月，*P*=0.04）。多因素分析，ECOG评分0分-1分、基线CYFRA21-1正常水平、治疗1月后CEA下降患者更能从EGFR-TKIs治疗中获益（表 3）。

**1:**
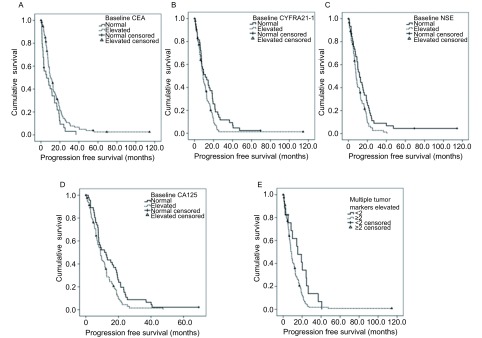
肿瘤标志物与EGFR-TKIs治疗PFS相关性。单因素分析中，基线CEA水平升高者EGFR-TKIs治疗PFS明显长于CEA正常者（中位PFS 9.8个月*vs* 5.9个月，*P*=0.027）（A），而基线NSE（B）、CYFRA21-1（C）、CA125（D）水平升高的患者较正常水平患者PFS明显缩短（中位PFS分别是7.9个月*vs* 11.5个月，*P*=0.015；9.0个月*vs* 11.4个月，*P*=0.029；9.0个月*vs* 11.5个月，*P*=0.023）。而（E）多项肿瘤标志物升高（≥2）的患者PFS明显缩短（中位PFS 8.1个月*vs* 15.3个月，*P*=0.03）。 Correlation between serum tumor markers and PFS of EGFR-TKIs. In the univariate analysis, PFS significantly prolonged in patients with elevated baseline CEA than those with normal CEA (mPFS 9.8 mo *vs* 5.9 mo, *P*=0.027) (A). While, to the opposite, PFS of patients with elevated baseline NSE (B), CYFRA21-1 (C) and CA125 (D) was significantly shorter than those with normal level of tumor markers (mPFS 7.9 mo *vs* 11.5 mo, *P*=0.015; 9.0 mo *vs* 11.4 mo, *P*=0.029; 9.0 mo *vs* 11.5 mo, *P*=0.023, respectively). Patients with multiple tumor markers elevated (≥2) progressed sooner than others (mPFS 8.1 mo *vs* 15.3 mo, *P*=0.03)(E).

**3:** 多因素分析各项因素与PFS的关系 Multivariate analysis of the relationship between various factors and PFS

Variate	PFS		OS	
	OR (95% CI)	*P*	OR (95% CI)	*P*
ECOG score	5.230 (1.94-14.1)	0.001	2.620 (1.057-6.454)	0.038
Metastatic sites	0.429 (0.174-1.06)	0.067	0.656 (0.175-2.459)	0.532
Mutation site	1.17 (0.409-3.326)	0.774	8.560 (2.708-27.055)	< 0.001
Baseline CEA	0.443 (0.065-3.000)	0.405	0.190 (0.024-1.495)	0.115
Baseline NSE	2.680 (0.805-8.939)	0.108	4.121 (0.79-21.502)	0.093
Baseline CYFA21-1	3.057 (0.974-9.598)	0.05	2.367 (0.396-14.149)	0.345
Baseline CA125	1.451 (0.567-3.711)	0.437	3.135 (0.731-13.444)	0.124
Baseline CA199	0.903 (0.256-3.192)	0.875	1.580 (0.378-6.597)	0.531
CEA decline in 1m	0.333 (0.12-0.92)	0.034	0.302 (0.093-0.977）	0.046
Multiple elevated markers	0.364 (0.077-1.715)	0.201	0.442 (0.068-2.856)	0.391
PFS: progression free survival; OS: overall survival.				

与总生存的相关性分析发现，基线CEA水平升高与否与总生存无关（中位OS 46.9个月*vs* 42.8个月，*P*=0.77），CYFRA21-1、CA125升高者总生存明显缩短（中位OS分别为25.1个月*vs* 52.5个月，*P*=0.003；22.7个月*vs* 55.0个月，*P* < 0.001）（见图 2A、图 2B）；多项肿瘤标志物升高者OS明显缩短（中位OS 24.6个月*vs* 75.5个月，*P*=0.005）（图 2C）。多因素分析显示，OS仅与ECOG分级（0分-1分）、*EGFR*突变位点以及治疗后1月CEA下降有关（表 3）。

**2:**
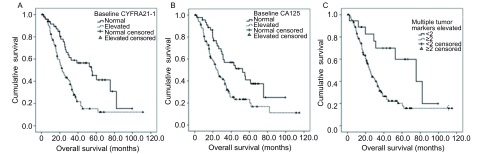
肿瘤标志物与OS相关性。单因素分析，CYFRA21-1（A）、CA125（B）升高的患者总生存明显缩短（中位OS分别为25.1个月*vs* 52.5个月，*P*=0.003；22.7个月*vs* 55.0个月，*P* < 0.001），而多项肿瘤标志物升高患者OS明显缩短（中位OS 24.6个月*vs* 75.5个月，*P*=0.005）（C）。 Correlation between serum tumor markers and OS. In the univariate analysis, OS significantly prolonged in patients with normal baseline CYFRA21-1 (A) and CA125 (B) (mOS 25.1 mo *vs* 52.5 mo, *P*=0.003; 22.7 mo *vs* 55.0 mo, *P* < 0.001, respectively). Patients with multiple tumor markers elevated survived longer (mOS 24.6 mo *vs* 75.5 mo, *P*=0.005)(C).

### 2.4 肿瘤标志物与二代测序结果

共13例患者接受了组织或外周血、胸水的二代测序，其中3例患者疗前肿瘤标志物中有5项升高（A组），4例仅有2项以下肿瘤标志物升高（B组）。A组测序结果提示存在4个-7个基因变异位点，包括EGFR多个位点的突变、TP53突变、MLL3突变、DH2 R172G突变、CTNNB1 S37C突变、MDM2拷贝数变异、CDK4拷贝数变异，而B组仅存在2个-3个基因变异位点，包括*EGFR*突变、*TP53*、*APC*突变。两组的PFS分别为（13.3±8.0）个月（A组）、（18.4±14.6）个月（B组）（表 4）。

**4:** 肿瘤标志物与基因变异的关系 Correlationship between tumor markers and gene variation

Group	Patient	Gene variation	Number of gene variations	Elevated tumor markers	Number of elevated tumor markers	PFS (months)
A	1	POM121L12, UNC13A	2	CEA, CYFRA21-1	2	42.8
A	2	EGFR L858R, TP53	2	CEA, CA199	2	6.53
A	3	EGFR 19 exon del, TP53	2	CA125	1	5.67
A	4	EGFR L858R, TP53, APC	3	CEA, CYFRA21-1, CA125	2	16.3
B	7	EGFR19/T790M/c.2389T > A p.C797S/ c.2390G > C p.C797S	4	CEA, NSE, CYFRA21-1, CA125, CA199	5	11.37
B	8	EGFR L858R, T790M, IDH2R172G and CTNNB1 S37C mutation, MDM2 and CDK4 amplification	7	CEA, NSE, CYFRA21-1, CA125, CA199	5	22.17
B	9	EGFR G719S/D837Y/L861Q, TP53, MLL3	5	CEA, NSE, CYFRA21-1, CA125, CA199	5	6.43
	10	None	0	CEA, NSE, CYFRA21-1	3	12.4
	11	EGFR L858R/T790M	2	CEA, NSE, CA125, CA199	4	2.1
	12	EGFR 19 exon del, TP53	2	CEA, CYFRA21-1, CA125	3	11.07
	13	PTEN, EGFR 19 exon del, TP53	3	CEA, CYFRA21-1, CA125	3	NA
	5	EGFRL858R, TP53	2	CEA, SCC, CYFRA21-1, CA125	4	8.8
	6	EGFR L858R/T790M, ERBB2, TP53	4	CEA, CYFRA21-1, CA125	3	14.33
NA: not available.						

## 3 讨论

目前，EGFR-TKIs因其疗效、生存以及不良反应上的优势已广泛应用于*EGFR*基因突变型NSCLC^[3-7]^，尤其是肺腺癌，已作为一线标准治疗列入相关指南^[13, 14]^。但临床实践中，并非所有*EGFR*敏感突变者均能从TKIs治疗中获益，因此，临床上需要更多方便易得的指标在*EGFR*突变患者中寻找优势人群。

既往研究^[15, 16]^发现，CEA水平与肺癌*EGFR*突变呈正相关，且高水平CEA更倾向于出现19外显子突变^[16]^，而相较于其他位点，19外显子突变可能对EGFR-TKIs反应更佳^[17]^，推测CEA升高者可能对EGFR-TKIs疗效更好。Okamoto等^[18]^早就发现，血清高水平CEA是晚期NSCLC接受吉非替尼治疗的良好预后因子。国内赵玲娣等^[19]^对166例接受吉非替尼治疗的晚期NSCLC患者进行分析，发现CEA水平升高的患者疾病控制率以及PFS都明显提高。金波等^[20]^也得出了类似的结论，同时发现一定范围内CEA水平越高，EGFR-TKIs疗效越好。

本中心发现，*EGFR*突变型肺鳞癌对EGFR-TKIs不敏感，甚至耐药^[21]^，对鳞癌患者进行TKIs疗效的相关性分析意义有限。基于此，本研究筛选了*EGFR*突变型晚期肺腺癌患者，分析疗前肿瘤标志物与一线EGFR-TKIs疗效及生存的相关性，发现疗前CEA升高者疗效更好、PFS延长，与既往NSCLC中结果一致。突变型NSCLC可通过EGFR途径下游的AKT及STAT被激活，引起抗凋亡活动增加，从而导致抗凋亡相关蛋白CEA的表达增加引起血清CEA升高^[18]^，所以CEA升高者可能*EGFR*突变丰度更高，而*EGFR*突变丰度往往与TKIs疗效呈正相关^[22]^，上述结果可能与此相关。

本研究中有58例患者治疗后1个月再次检测了肿瘤标志物，发现CEA、CA125下降明显者TKIs疗效越好，这一点与许阳等^[23]^的结论不谋而合，他们也发现EGFR-TKIs治疗后CEA下降的患者更能从治疗中获益。金波等^[20]^更详细地根据治疗后CEA下降的情况将患者分组，发现CEA直接下降者较CEA先升后降者疗效更好。而本研究由于疗后CEA检测时间点不统一，并未根据肿瘤标志物变化规律进行具体分析。

除了CEA，本研究还对其他肿瘤标志物与EGFR-TKIs疗效进行了分析。其中，尽管与TKI疗效无关，CYFRA21-1（主要由肿瘤细胞坏死导致角蛋白释放产生）却与PFS、OS呈负相关。部分学者如Barlesi、Tanaka等^[10, 24]^，认为CYFRA21-1升高者接受EGFR-TKIs治疗后PFS更短（7. 5个月*vs* 13. 3个月），OS并无差异。但也有学者^[25]^认为CYFRA21-1与EGFR-TKIs治疗后的生存并无相关。这些研究结果的差异可能与样本量小、入组标准以及取血点不同有一定关系。另外，作为杂交瘤肿瘤家族中的一种肿瘤标记物，CA125在NSCLC中的作用越来越受到重视。本研究中，CA125升高提示生存获益小，而治疗后CA125下降明显者更能从TKIs治疗中获益。既往研究较少对CA125与TKIs疗效进行分析，部分研究认为CA125升高者3年生存率更高^[26]^，而部分研究则认为两者并无相关性^[12]^。一项基础研究表明TKIs和EGFR单抗可以抑制癌细胞CA125的表达，但对培养液中CA125浓度没有影响，所以血清CA125水平不一定能反映TKIs真实的疗效^[27]^。所以，对于CA125、CYFRA21-1等其他肿瘤标志物对EGFR-TKIs治疗的影响，目前尚不明确。

另外，本研究还对多项肿瘤标志物升高者进行分析，发现其与生存呈负相关，而与疗效无关，但由于各项肿瘤标志物对TKIs疗效影响不一，这种缺少权重的进行绝对分类可能引起结果偏差，日后的研究可能需要对多项肿瘤标志物进行更具体的组合分类。

本研究中部分患者进行了组织或外周血二代测序，结果发现，基因变异越多的患者更有可能出现多项肿瘤标志物升高，可能与基因变异如*EGFR*、*TP53*突变导致肿瘤细胞增殖，引起相关蛋白如CEA、CA125等表达升高有关。但由于例数过少、变异基因宽泛，两者异质性是否相关仍未可知，未来仍需要大量样本的病例及基础研究进行分析。

综上，本研究认为，基线CEA水平以及治疗过程中CEA的变化可以预测肺腺癌患者一线接受EGFR-TKI治疗的疗效，而治疗前高水平CYFRA21-1以及CA125则预示着生存期缩短。然而，由于病例数及回顾性研究的限制，未来需要更多前瞻性临床研究以及基于基因及分子基础的研究来证实。
